# An Outbreak of Lymphocutaneous Sporotrichosis among Mine-Workers in South Africa

**DOI:** 10.1371/journal.pntd.0004096

**Published:** 2015-09-25

**Authors:** Nelesh P. Govender, Tsidiso G. Maphanga, Thokozile G. Zulu, Jaymati Patel, Sibongile Walaza, Charlene Jacobs, Joy I. Ebonwu, Sindile Ntuli, Serisha D. Naicker, Juno Thomas

**Affiliations:** 1 National Institute for Communicable Diseases, a Division of the National Health Laboratory Service, Johannesburg, South Africa; 2 University of the Witwatersrand, Johannesburg, South Africa; 3 University of Pretoria, Pretoria, South Africa; University of California San Diego School of Medicine, UNITED STATES

## Abstract

**Background:**

The largest outbreak of sporotrichosis occurred between 1938 and 1947 in the gold mines of Witwatersrand in South Africa. Here, we describe an outbreak of lymphocutaneous sporotrichosis that was investigated in a South African gold mine in 2011.

**Methodology:**

Employees working at a reopened section of the mine were recruited for a descriptive cross-sectional study. Informed consent was sought for interview, clinical examination and medical record review. Specimens were collected from participants with active or partially-healed lymphocutaneous lesions. Environmental samples were collected from underground mine levels. *Sporothrix* isolates were identified by sequencing of the internal transcribed spacer region of the ribosomal gene and the nuclear calmodulin gene.

**Principal Findings:**

Of 87 male miners, 81 (93%) were interviewed and examined, of whom 29 (36%) had skin lesions; specimens were collected from 17 (59%). Sporotrichosis was laboratory-confirmed among 10 patients and seven had clinically-compatible lesions. Of 42 miners with known HIV status, 11 (26%) were HIV-infected. No cases of disseminated disease were detected. Participants with ≤3 years’ mining experience had a four times greater odds of developing sporotrichosis than those who had been employed for >3 years (adjusted OR 4.0, 95% CI 1.2–13.1). Isolates from 8 patients were identified as *Sporothrix schenckii* sensu stricto by calmodulin gene sequencing while environmental isolates were identified as *Sporothrix mexicana*.

**Conclusions/Significance:**

*S*. *schenckii* sensu stricto was identified as the causative pathogen. Although genetically distinct species were isolated from clinical and environmental sources, it is likely that the source was contaminated soil and untreated wood underground. No cases occurred following recommendations to close sections of the mine, treat timber and encourage consistent use of personal protective equipment. Sporotrichosis is a potentially re-emerging disease where traditional, rather than heavily mechanised, mining techniques are used. Surveillance should be instituted at sentinel locations.

## Author Summary

Sporotrichosis is a neglected tropical fungal disease. The subcutaneous form occurs following traumatic inoculation of organic matter contaminated with the fungus through the skin. Between 1938 and 1947, the Witwatersrand gold mines in South Africa were the site of the largest outbreak of sporotrichosis worldwide. Contamination of untreated timber by the fungus was thought to be associated with the outbreak though this epidemiologic link was never made definitively. For the first time in this setting, we conducted a detailed outbreak investigation to identify factors associated with sporotrichosis. The investigation identified 17 cases of sporotrichosis at a reopened South African gold mine. Approximately one in five miners had probable or confirmed sporotrichosis. Although genetically distinct species were isolated from clinical and environmental sources, it is highly likely that the source was contaminated soil and untreated wood underground. No cases occurred following recommendations to close sections of the mine, treat timber and encourage consistent use of personal protective equipment. The investigation uncovered a re-emerging disease in mines where traditional, rather than heavily mechanized, ore extraction techniques are used. Surveillance should be instituted at sentinel mining locations.

## Introduction

Sporotrichosis is a subcutaneous mycosis that usually occurs following traumatic inoculation of organic matter contaminated with thermally-dimorphic fungi within the *Sporothrix schenckii* species complex [1]. In South Africa, approximately 3300 miners were clinically diagnosed with sporotrichosis between 1938 and 1947 on the Witwatersrand [2–4]. Contamination of timber by the fungus was thought to be associated with the outbreak and *S*. *schenckii* sensu lato was not cultured from environmental sources despite several attempts (4). Subsequent to this, sporadic cases and small outbreaks of disease were reported [5]. The HIV epidemic in South Africa has not been associated with a concomitant increase in diagnosed cases of sporotrichosis and the prevalence has remained low [6].

In 2006, Marimon et al provided evidence that the pathogenic species, *S*. *schenckii* sensu lato was a species-complex comprising of several cryptic species [7]. By sequencing portions of the nuclear calmodulin (CAL), β-tubulin and chitin synthase genes of 60 isolates, most of clinical origin, at least six phylogenetic clades could be distinguished corresponding largely to the geographic source. Further work revealed three new closely-related cryptic species that were distinct from *S*. *schenckii* sensu stricto by phylogenetic analysis of the CAL gene: the hyper-virulent *Sporothrix brasilensis*, *Sporothrix globosa* and a third species that was initially only isolated from the environment and has more recently been isolated from human and animal cases, *Sporothrix mexicana* [8].

Following initial detection of two cases of laboratory-confirmed sporotrichosis at a reopened section of a gold mine in South Africa in 2011, an outbreak investigation was initiated. Here we describe the epidemiology of this outbreak as well as the species-level identification, antifungal susceptibility and genetic relatedness of clinical and environmental isolates obtained through the investigation.

## Methods

### Study site, design and active case-detection

The outbreak occurred among workers employed at a gold mine close to the town of Barberton in the sub-tropical north-eastern Lowveld area of South Africa. Approximately 2000 workers were employed at the mine-complex at the time of investigation. To identify eligible participants for a descriptive, cross-sectional study, a list of employees working at a reopened section of the mine was obtained. Participants were recruited over three days in August/September 2011 at the mine and informed consent was sought for interview and medical record review. Patients with lymphocutaneous lesions that were clinically-compatible with sporotrichosis were asked to provide informed consent to have specimens submitted for culture and/or to have non-identifying photographs taken of the lesions. A list of employees who had sought medical care and had been diagnosed with skin lesions of chronic duration (>1 month) was obtained from the mine’s occupational health clinic. Medical personnel at the nearby Barberton Hospital were also requested to report cases of suspected sporotrichosis.

### Case definitions

A confirmed case of sporotrichosis was defined as a person who was employed at the gold mine with a specimen that was positive on culture or histology for *S*. *schenckii*. A probable case was defined as an employee with a culture-negative but active or healed lymphocutaneous lesion that was clinically compatible with sporotrichosis.

### Data collection

A structured, interviewer-administered questionnaire was completed in English, isiZulu and siSwati for all eligible participants who consented. Workers were educated, interviewed and examined by the field team during working hours. Participants were examined for lymphocutaneous lesions or evidence of disseminated sporotrichosis by a team medical doctor (N.P.G, S.N. or J.T.). If a lesion that was clinically compatible with sporotrichosis was identified, an additional case investigation form was completed and a specimen was collected, if possible. The occupational health clinic records of cases with probable or confirmed sporotrichosis were reviewed.

### Data management and analysis

Data were entered, cleaned, verified and analysed using Epi Info version 3.3.2 (Centers for Disease Control and Prevention, Atlanta, GA) and STATA version 12.1 (StataCorp, College Station, TX). Categorical variables were compared using the chi-squared test or the Fisher’s exact test. Odds ratios and 95% confidence intervals (CI) were calculated using logistic regression. The multivariable logistic regression model was evaluated by stepwise addition of all variables that were significant at p <0.15 on univariate analysis. Two-sided p values of <0.05 were considered significant throughout.

### Collection of clinical and environmental specimens

Specimens (including punch biopsies, scrapings or swabs) were collected from participants with active or partially-healed lymphocutaneous lesions [5]. At the same time, environmental samples (including visible fungus, soil and wood scrapings) were collected in sterile containers from eight separate underground mine levels where miners had worked or were currently working; sample collection was not systematic.

### Laboratory methods

Briefly, clinical and environmental specimens were processed at a reference laboratory where isolates of *S*. *schenckii* sensu lato were identified using standard phenotypic methods [1]. Identification of fungal isolates that resembled *S*. *schenckii* by phenotypic methods was confirmed by sequencing of the internal transcribed spacer (ITS) region of the ribosomal gene, which allowed identification to the species complex level, and sequencing of the nuclear CAL gene, which allowed the isolates to be identified to the cryptic species level [7,9]. Minimum inhibitory concentrations (MICs) for amphotericin B, voriconazole, itraconazole and posaconazole were determined by broth dilution and Etest ((bioMérieux, Marcy ľEtoile, France) for the yeast phase. Consensus CAL gene sequences from the outbreak strains, ten reference strains and nine clinical strains unrelated to the outbreak were used to generate a dendrogram. Detailed laboratory methods are provided in S1 Text.

### Ethics review and permissions

Prior to the investigation, urgent written approval was obtained from the Human Research Ethics Committee (Medical) of the University of the Witwatersrand and the provincial department of health was consulted. Mine management gave permission and consulted with union representatives. Potential participants were provided with information about sporotrichosis and the investigation and were requested to provide written, informed consent.

## Results

### Case detection

**Fig 1:**
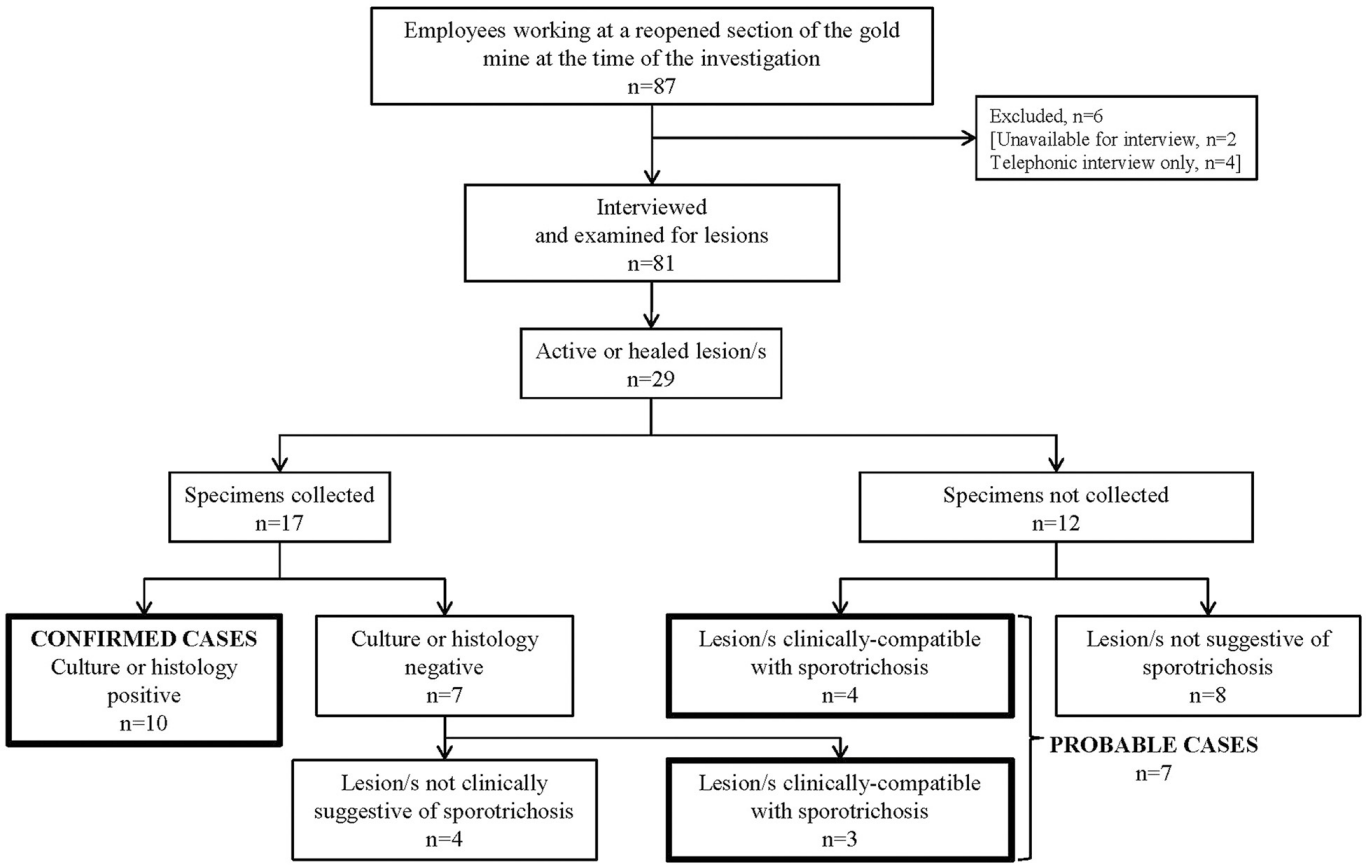
Flowchart of cases of confirmed and probable sporotrichosis detected at a reopened section of a gold mine by month, Barberton, 2009–2011, n = 17.

**Fig 2:**
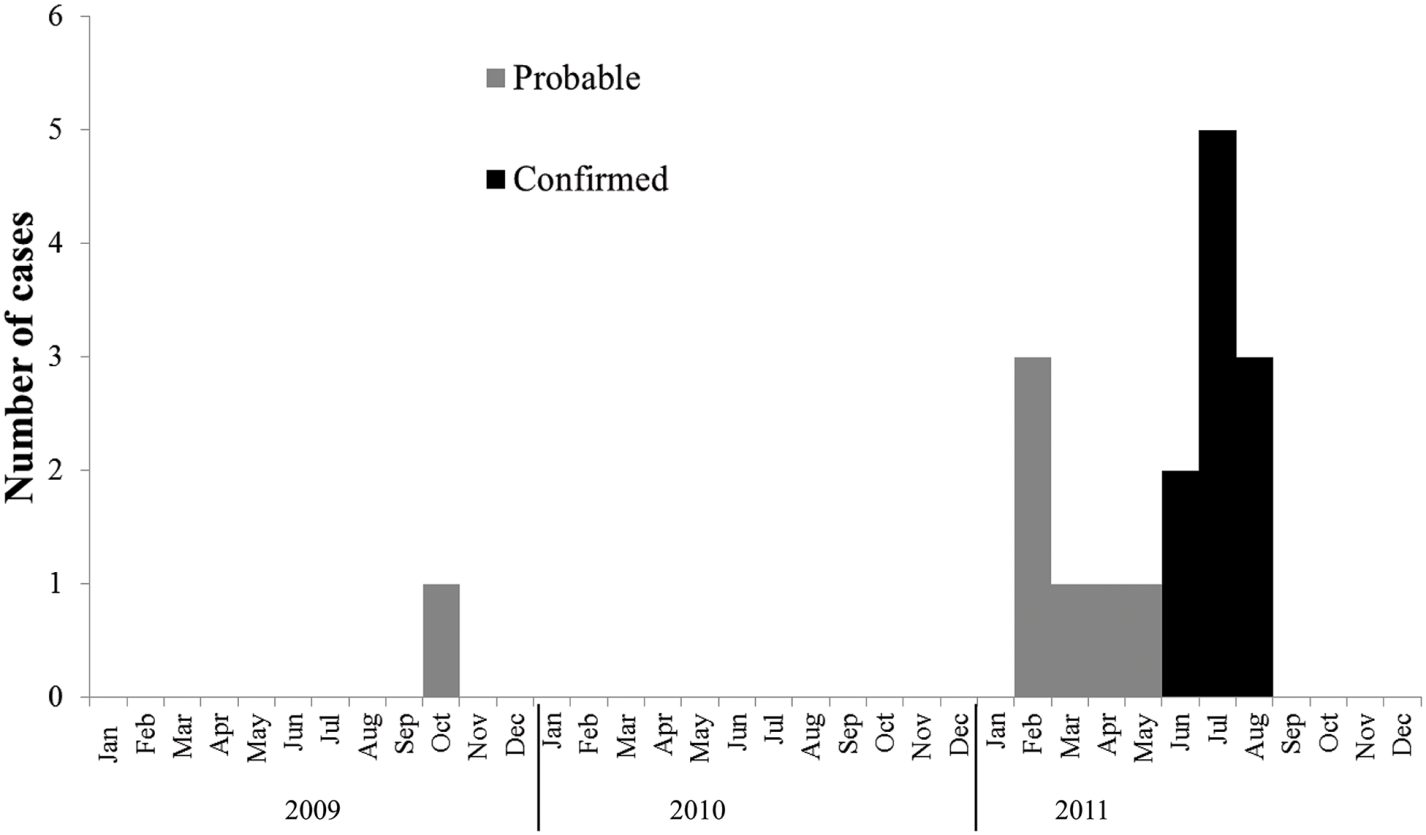
Epidemic curve with cases of confirmed and probable sporotrichosis detected at a reopened section of a gold mine by month, Barberton, 2009–2011, n = 17.

Eighty one of 87 male miners (93%) were interviewed and examined (Fig 1). Twenty-nine (33%) had active, partially-healed or healed skin lesions and specimens were collected from 17 (59%). Of these 17 patients, sporotrichosis was confirmed among 10 (eight with culture-confirmed disease and two with histologically-compatible disease). Specimens from the other seven were culture-negative; however, three patients met the case definition for probable sporotrichosis. Of the 12 participants with skin lesions from whom no specimens were collected, four patients also met the case definition for probable sporotrichosis. Seventeen participants were thus classified as having confirmed or probable sporotrichosis, i.e. 10 confirmed and seven probable, with a prevalence of 20% (17/81) (Fig 1). The epidemic curve illustrates that the outbreak may have been undetected for many months (Fig 2). A case of probable sporotrichosis with healed lesions in a classic distribution was reported with disease onset as early as October 2009.

### Demographic and clinical data

The median age of participants with and without sporotrichosis was similar (34 years (interquartile range (IQR), 25 to 48 years) vs. 34 years (IQR, 25 to 48 years); p = 0.8) (Table 1). More than a quarter of participants (23/81; 28%) were in the 25–29 year age group. Of 42 participants with known HIV infection status, 11 (26%) were HIV-infected. Miners with confirmed or probable sporotrichosis were no more likely to be HIV-infected than miners without disease (unadjusted OR1.9, 95% CI 0.4–10.0; p = 0.42). Only six participants reported other underlying medical conditions: diabetes mellitus (n = 2), previous tuberculosis (n = 2), a history of cancer (n = 1) and chronic lung disease (n = 1). All participants worked underground. Thirty per cent (25/81) had previously worked at another mine in Barberton and 73% (59/81) were general mine workers. Thirty five participants (43%) had worked at the mine for ≤3 years. The majority of participants reported that that they currently worked on mine level 34 (17/81; 25%). A large proportion of miners recalled minor skin injuries and direct skin contact with soil and wood. Almost all participants reported that they used personal protective equipment (PPE) (including masks, goggles, helmets, gloves, boots and overalls) consistently, that they showered immediately after a shift and that they laundered their overalls more than once a week. On multivariable analysis, participants who had been employed for ≤3 years were four times more likely to have either confirmed or probable sporotrichosis (adjusted OR 4.0, 95% CI 1.2–13.1) (Table 1).

**Table 1:** Comparison of miners with and without confirmed or probable sporotrichosis at a reopened section of a gold mine, Barberton, n = 81.

Characteristic		Miners with confirmed or probable sporotrichosis (n = 17)	Miners without confirmed or probable sporotrichosis (n = 64)	p-value	Unadjusted odds ratio (95% CI)	p-value	Adjusted odds ratio (95% CI)	p-value
Median age, IQR (years)		34 (25–40)	34 (25–48)					
Age category (years)	20–24	1/17 (6)	3/64 (5)	0.61	Reference	0.67		
	25–29	5/17 (29)	18/64 (28)		0.83 (0.07–9.86)			
	30–34	4/17 (23)	15/64 (23)		0.80 (0.06–9.92)			
	35–39	3/17 (18)	14/64 (22)		0.64 (0.05–8.52)			
	40–44	1/17 (6)	8/64 (12)		0.38 (0.17–8.10)			
	45–49	3/17 (18)	3/64 (5)		3.00 (0.19–47.96)			
	50–54	0/17 (0)	3/64 (5)		Unspecified			
Previous work at other mines	No	10/17 (59)	46/64 (72)	0.30	Reference	0.31		
	Yes	7/17 (41)	18/64 (28)		1.78 (0.59–5.42)			
**Duration working at mine (years)**	**> 3**	**5/17 (29)**	**41/64 (64)**	**0.01**	**Reference**	**0.01**	**Reference**	**0.02**
	**≤ 3**	**12/17 (71)**	**23/64 (36)**		**4.29 (1.34–13.67)**		**4.00 (1.22–13.13)**	
Occupational category	Supervisor	1/17 (6)	13/64 (20)	0.26	Reference	0.21		
	Specialist worker	1/17 (6)	7/64 (11)		1.85 (0.10–34.43)			
	General worker	15/17 (88)	44/64 (69)		4.43 (0.53–36.80)			
Hours worked underground per day**	≤ 5 hours	1/16 (6)	7/61 (12)	0.54	Reference	0.52		
	> 5 hours	15/16 (94)	54/61 (88)		1.95 (0.22–17.06)			
Current mine level**	Surface	1/16 (6)	1/51 (2)	0.17	Reference	0.90		
	All levels	1/16 (6)	7/51 (13)		0.14 (0.04–4.61)			
	Level 14	2/16 (13)	8/51 (15)		0.25 (0.01–5.98)			
	Level 16	4/16 (24)	10/51 (19)		0.40 (0.19–8.07)			
	Level 30	0/16 (0)	7/51 (13)		Unspecified			
	Level 32	2/16 (13)	0/51 (0)		Unspecified			
	Level 34	4/16 (25)	13/51 (25)		0.31 ((0.02–6.11)			
	Level 38	2/16 (13)	7/51 (13)		0.29 (0.12–6.91)			
HIV-infected**	No	5/8 (63)	26/34 (76)	0.42	Reference	0.43		
	Yes	3/8 (37)	8/34 (24)		1.95 (0.38–10.01)			
Other underlying diseases*	No	16/17 (94)	59/64 (92)	0.79	Reference	0.78		
	Yes	1/17 (6)	5/64 (8)		0.74 (0.08–6.76)			
Reported cuts or scratches on exposed areas	No	7/17 (41)	32/64 (50)	0.52	Reference	0.51		
	Yes	10/17 (59)	32/64 (50)		1.42 (0.48–4.21)			
Reported wood splinter injury or skin contact with wood	No	4/17 (24)	10/64 (16)	0.44	Reference	0.45		
	Yes	13/17 (76)	54/64 (84)		0.60 (0.16–2.23)			
Reported skin contact with soil	No	1/17 (6)	9/64 (14)	0.36	Reference	0.33		
	Yes	16/17 (94)	55/64 (86)		2.62 (0.31–22.25)			
Frequent use of personal protective equipment^†^	No	1/17 (6)	6/64 (9)	0.65	Reference	0.63		
	Yes	16/17 (94)	58/64 (91)		1.66 (0.19–14.76)			
Shower immediately after shift	No	0/17 (0)	2/64 (3)	0.46	Unspecified^††^			
	Yes	17/17 (100)	62/64 (97)					
Launder overalls more than once per week**	No	0/15 (0)	3/61 (5)	0.38	Unspecified^††^			
	Yes	15/15 (100)	58/61 (95)					
Reported outdoor activities outside of work	No	4/17 (24)	34/64 (53)	0.03	Reference	0.03	Reference	0.06
	Yes	13/17 (76)	30/64 (47)		3.68 (1.08–12.51)		3.4 (0.96–12.00)	

*Denominators are less than column total because of missing data;

**Previous or current tuberculosis, malignancy, diabetes or chronic lung disease;

^†^Reported use of gloves, boots, masks, goggles, helmets and overalls as “always” or “most of the time”;

^††^Not included in the multivariable model.

### Cases of sporotrichosis

**Fig 3:**
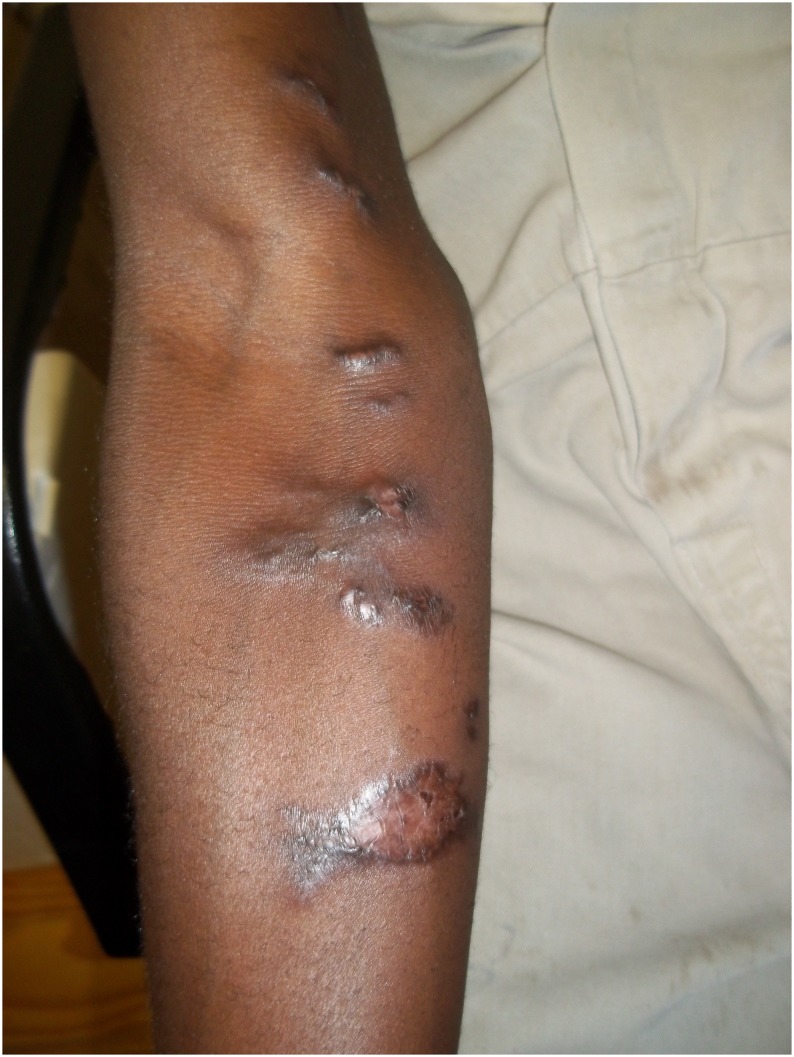
A patient with probable sporotrichosis of the upper limb with a chain of healed lesions in a classic lymphocutaneous distribution.

Specimens were collected from 17 participants with active or partially-healed lesions (Fig 2). The majority (14/17) had received itraconazole before specimen collection (Table 2). Most specimens were scrapings of crusts that had formed on partially-healed skin lesions. Three patients with clinically-compatible active/healed lesions were classified as probable cases because there was no laboratory evidence of disease, despite specimen submission (Fig 3). Four other patients with no laboratory evidence were classified as participants without sporotrichosis because their lesions were not clinically compatible. All cases with confirmed sporotrichosis had partially-healed, multiple lymphocutaneous lesions (<5 lesions) and had received itraconazole, whereas only three of the 7 cases with probable sporotrichosis had active lesions and only four had started itraconazole (Table 2). Three of ten confirmed cases reported having had a cut or scratch before their lesions appeared. The majority of skin lesions (14/17; 82%) were nodules and/or ulcers. HIV infection status was known for only four patients with confirmed sporotrichosis; three of the four were HIV-infected. No patients had any clinical evidence of disseminated sporotrichosis.

**Table 2:** Comparison of miners with confirmed and probable sporotrichosis at a reopened section of a gold mine, Barberton, n = 17.

Characteristic		Cases of confirmed sporotrichosis* (N = 10)	Cases of probable sporotrichosis* (N = 7)
Location of skin lesions	Head and neck	1/10 (10)	1/7 (14)
	Upper limb/s	8/10 (80)	6/7 (86)
	Lower limb/s	1/10 (10)	0/7 (0)
	Trunk	0/10 (0)	0/7 (0)
Type of skin lesion	Nodule	0/10 (0)	1/7 (14)
	Ulcer	1/10 (10)	4/7 (57)
	Nodule and ulcer	7/10 (70)	1/7 (14)
	Nodule, ulcer, draining pus	1/10 (10)	0/7 (0)
	Ulcer and verrucous lesion	1/10 (10)	0/7 (0)
	Ulcer and plaque	0/10 (0)	1/7 (14)
Palpable lymphatic vessels		3/10 (30)	0/7 (0)
Local lymphadenopathy		5/10 (50)	1/7 (14)
Active skin lesion (vs. healed)		10/10 (100)	3/7 (43)
Median duration of skin lesions, IQR (days)		49 (21–61)	181 (123–241)
Multiple skin lesions		10/10 (100)	6/7 (86)
Number of skin lesions	≤ 5 lesions	10/10 (100)	4/7 (57)
	> 5 lesions	0/0 (0)	3/7 (43)
On itraconazole treatment at the time of clinical examination		10/10 (100)	4/7 (57)
Median time from lesion appearance to initiation of itraconazole treatment, IQR (days)		35 (8–51)	153 (102–219)
Reported cut or scratch before lesion		3/10 (30)	2/7 (29)

*None of the patients had clinical evidence of disseminated disease.

### Observation of surface and underground conditions and environmental sample results

Four members of the outbreak team (N.P.G., S.W., T.G.Z. and N.M.) visited three underground levels, two of which were closed for work at the time of the visit (levels 30 and 32). Underground conditions were extremely uncomfortable and the heat, humidity and poor ventilation were most marked in the closed levels. Rotting timber poles were stacked to the side of tunnels and visible growth of a white filamentous fungus was noted on timber and in the water-logged or moist soil. Drainage was poor in the closed levels with stagnant pools of water collecting in the tunnels. Wooden ladders had rotted away and when descending steep slopes and chutes, it was difficult to avoid contact with visibly-contaminated wood and soil even while wearing PPE. Conditions were more comfortable on open level 34. However, growth of a white fungus was also visible on soil and on rotting timber piles that had been caged off. PPE was not consistently used by a group of partially-dressed miners who were briefly observed at rest and at work. The process of ore extraction was manual rather than mechanised and workers were exposed to flying shards of rock and chunks of soil as they worked. On direct questioning, miners reported leaving work garments underground. Photographs, taken at the sites of specimen collection, showed visible growth of a white fungus at all collection sites. A surface visit was also conducted near the mine shaft and stacks of untreated timber were observed. No surface environmental samples were collected.

### Laboratory identification of isolates

Twenty three specimens (13 lesion scrapings and 10 pus swabs in transport medium) were collected from 17 patients. Eleven *S*. *schenckii* sensu lato isolates were cultured from specimens of eight patients with confirmed sporotrichosis; two patients had fungi isolated from both scrapings and pus swabs. Clinical isolates were confirmed as *S*. *schenckii* sensu stricto by sequencing (Table 3). Fungal isolates were also cultured from an environmental sample from a closed section (level 30) and from four samples from an open section (level 34). In contrast, although these isolates (from 5 samples) were identified as *S*. *schenckii* sensu lato by ITS sequencing, CAL gene sequencing of isolates (from 4 samples where DNA was still available) confirmed the identification as *S*. *mexicana*. The mean mould-phase colony diameter of the *S*. *schenckii* sensu stricto isolates was less than the mean colony diameter of the *S*. *mexicana* isolates (20.9 mm ± 11.4 mm vs. 50.0 mm ± 14.1 mm). The yeast phase of all isolates grew at 37°C. The *S*. *schenckii* sensu stricto isolates developed colony pigmentation much earlier than the *S*. *mexicana* isolates; the latter became melanised after 3 to 6 weeks of incubation at room temperature and ambient light.

**Table 3:** Minimum inhibitory concentrations for the yeast phase of *Sporothrix schenckii* complex isolates, n = 13.

Isolate number	Source	Species-level identification*	Minimum inhibitory concentration (mg/L):							
			AMB BMD	AMB Etest	VRC BMD	VRC Etest	POS BMD	POS Etest	ITC BMD	ITC Etest
38.1^†^	Clinical	*Sporothrix schenckii*	4	>32	0.25	2	0.5	0.75	0.5	0.75
41.1^†^	Clinical	*Sporothrix schenckii*	>8	>32	0.5	2	0.5	0.25	0.5	0.75
53	Clinical	*Sporothrix schenckii*	1	>32	0.008	2	0.008	0.19	0.015	2
55	Clinical	*Sporothrix schenckii*	Contaminated							
57	Clinical	*Sporothrix schenckii*	>8	1	0.25	1.5	0.5	0.094	0.25	0.5
60	Clinical	*Sporothrix schenckii*	>8	>32	0.5	4	0.5	0.19	0.5	4
82	Clinical	*Sporothrix schenckii*	>8	>32	0.5	2	0.5	0.5	0.5	4
85	Clinical	*Sporothrix schenckii*	>8	4	1	4	0.25	0.25	0.12	0.019
1	Environmental	*Sporothrix mexicana*	>8	>32	>8	>32	>8	>32	>16	>32
7	Environmental	*Sporothrix* species	>8	>32	4	>8	>8	>32	>16	>32
8	Environmental	*Sporothrix mexicana*	2	>32	0.25	1.5	0.5	>32	0.5	>32
10	Environmental	*Sporothrix mexicana*	>8	>32	>8	>32	>8	>32	>16	>32
12	Environmental	*Sporothrix mexicana*	>8	>32	4	>32	>8	>32	>16	>32

*Species-level identity was determined by sequencing of the nuclear calmodulin gene;

^†^multiple isolates from the same patient were not tested (i.e. isolates 38.2, 41.2 and 41.3);

abbreviations: AMB: amphotericin B; VRC: voriconazole; POS: posaconazole; ITC: itraconazole; BMD: broth microdilution test.

### Antifungal susceptibility results

The amphotericin B MICs were high for almost all clinical isolates (Table 3). Broth microdilution MICs were low for itraconazole, posaconazole and voriconazole with an MIC_50_ of 0.5 mg/L for all three agents. In general, the five environmental isolates exhibited very high MICs to all tested antifungals (greater than the maximum tested antifungal concentration).

### Phylogenetic analysis

**Fig 4:**
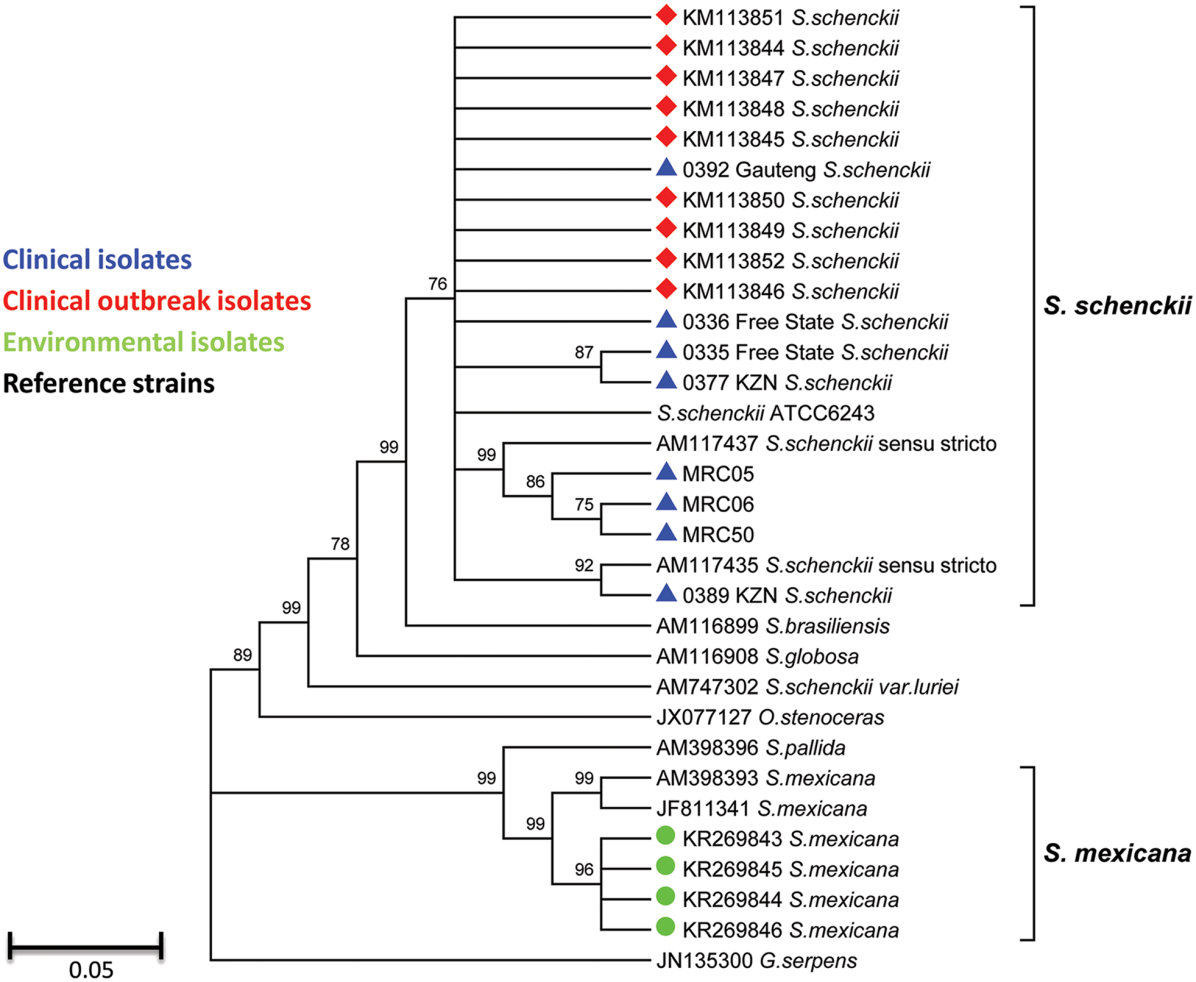
Phylogenetic analysis of the partial calmodulin gene sequences from 10 clinical outbreak isolates, 5 environmental outbreak isolates, 9 unrelated clinical strains and 10 reference strains.

Thirty four partial calmodulin gene sequences were included in the alignment, 24 of which were generated in this study. The sequences of five environmental isolates (from 4 samples) clustered most closely with those of the *S*. *mexicana* type strain (Fig 4). The sequences of ten clinical isolates (from eight patients) clustered most closely with those of the *S*. *schenckii* sensu stricto type strain, the *S*. *schenckii* ATCC 6243 strain and the South African *S*. *schenckii* sensu stricto clinical isolates. The clinical strains were clearly separated from the *S*. *brasilensis* and *S*. *globosa* type strains.

## Discussion

An outbreak investigation identified 17 cases of sporotrichosis among gold miners at a reopened section of a mine in South Africa. Approximately one in five miners working in that section had probable or confirmed sporotrichosis. The outbreak may have been undetected for many months because a case of probable disease was reported with an onset two years preceding the investigation. The majority had started antifungal treatment for sporotrichosis at the time of the investigation. CAL gene sequencing of ten clinical and five environmental isolates showed that the two groups of isolates were genetically distinct; the closely-related clinical isolates were identified as *S*. *schenckii* sensu stricto while the environmental isolates were identified as *S*. *mexicana*. This is the first time that *S*. *mexicana* has been isolated and identified in South Africa. The *S*. *mexicana* isolates had almost uniformly elevated MICs to all tested antifungal agents while the *S*. *schenckii* sensu stricto isolates were relatively more susceptible to itraconazole, posaconazole and voriconazole *in vitro*.

### Clinical disease

Though the prevalence of sporotrichosis among miners was very high, this was probably a minimum estimate because some cases may have resolved spontaneously without confirmation of diagnosis [4,5]. All miners with confirmed/probable sporotrichosis had lymphocutaneous disease. Most patients had lesions on exposed areas such as the head, neck and upper limbs where soil may have come into contact with skin, or wood splinter injuries may have occurred. Several patients had upper limb lesions that were located at or above the point where their long protective gloves ended. Anecdotally, other patients who had developed lesions on their heads or necks reported carrying timber poles on their shoulders. Among cases with confirmed disease, all had multiple lesions that were either nodular or ulcerating; a few had palpably thickened lymphatic vessels and half had local lymphadenopathy. The vast majority of cases with probable disease also had multiple lesions; this is not surprising because the clinical pattern of a healed chain of lesions along the lymphatic vessels is easier to recognise than a healed single lesion. All miners with sporotrichosis had a clear response to itraconazole at the time of clinical examination. The clinical findings are consistent with a previous report from the mines on the Witwatersrand and from other case series in South Africa [2,4,5]. Even though more than a quarter of miners with known HIV infection status were HIV-infected, none had evidence of disseminated disease. HIV-infected patients with preserved T cell-mediated immunity may respond to infection in the same way as immunocompetent patients. The interviewed miners with HIV infection were employed in physically-gruelling manual labour and we speculate that they were less likely to be severely immunosuppressed. We found no association between occupational category of underground worker and disease [4]. On multivariable analysis, only duration of employment at the mine for a period ≤ 3 years was significantly associated with confirmed or probable disease. Although this is counter-intuitive, we speculate that less-experienced workers may have used PPE less regularly or incorrectly and may have incurred more frequent minor injuries and contact with wood and soil. On univariate analysis, reporting of outdoor activities outside of work was significantly associated with sporotrichosis; however, this association did not remain significant on multivariable analysis.

### Recommendations made shortly after the outbreak investigation

Improved ventilation, through engineering controls such as extraction fans to reduce humidity and heat, was recommended for open levels. It was also recommended that miners not be deployed to work in areas with visible fungal contamination and that visibly-contaminated timber be isolated from working areas by caging or removed. It was also recommended that all new timber be treated with tar. PPE was recommended and additional protective arm-guards extending from the forearm to above the elbow were used by workers when handling visibly-contaminated material. We also recommended that workers be encouraged not to leave overalls underground because *S*. *schenckii* complex is able to grow on various clothing fabrics [5]. Clothing was recommended to be laundered regularly and workers to be supplied with more than one set of overalls. We recommended that a high index of clinical suspicion be maintained, that patients have appropriate specimens collected to confirm the diagnosis and that patients with lymphocutaneous disease be treated with itraconazole. Itraconazole was made available at the mine clinic and at the local district hospital. To date, no additional cases have been reported.

### Laboratory identification and antifungal susceptibility

The phenotypic characteristics of the clinical and environmental isolates were consistent with previous reports [8], though it is notable that *S*. *mexicana* mould-phase colonies melanised very slowly, possibly due to their underground origin. In line with previous reports, the clinical outbreak isolates of *S*. *schenckii* sensu stricto had relatively low itraconazole and posaconazole BMD MICs [10–12]. We found MICs close to or at the maximum tested concentration for amphotericin B for the clinical outbreak isolates possibly because we tested the yeast form. None of the patients required treatment with amphotericin B and so we are unable to comment on clinical outcomes related to use of this agent. We also determined lower voriconazole BMD MICs for the clinical isolates than have been reported previously [10,11]. Again, this difference may have been as a result of the test method because the Etest generated consistently higher voriconazole MICs, more in line with previous reports. The environmental *S*. *mexicana* strains had almost uniformly elevated MICs to all tested antifungal agents [11].

### Source of the outbreak

While we were able to confirm the hypothesis that *S*. *schenckii* was the cause of this outbreak, we were unable to provide definitive evidence for the environmental source. With isolation of *S*. *schenckii* sensu lato from both clinical and environmental sources, it had initially seemed certain that the outbreak was caused by exposure of miners to the contaminated wood and soil underground. Due to several limitations of this study, we still believe that miners likely developed disease after exposure to fungi growing in underground mine levels. First, environmental sampling was limited to only eight underground levels and samples were also not collected from the surface. It is possible that multiple pathogenic species, including *S*. *schenckii* sensu stricto and *S*. *mexicana*, had established an ecological niche in the underground levels and that the niches containing species other than the more rapidly-growing *S*. *mexicana* were not sampled, e.g. areas where visible white fluffy mould was not growing. Second, although we made an effort to select all colonies that resembled *S*. *schenckii* sensu lato and were cultured from the environmental specimens, some colonies may have been missed. Although several colony phenotypes were observed on the original plates from some of the clinical and environmental specimens, we did not identify all colony phenotypes to species-level by CAL gene sequencing. *S*. *mexicana* is a less virulent species that has only been isolated from a few human and animal cases [13]. It is also possible that our outbreak investigation did not detect rarer clinical cases of *S*. *mexicana* infection. While we were unsuccessful, other investigators have established clearer links between clinical and environmental strains [9,14–17].

### Conclusions

*S*. *schenckii* sensu stricto was identified as the causative pathogen in a group of miners with lymphocutaneous disease at a South African gold mine. Although genetically distinct species were isolated from clinical and environmental sources, it is likely that the source was contaminated soil and untreated, rotting wood in the underground mine levels. Sporotrichosis is a potentially re-emerging disease in areas of South Africa where traditional, rather than heavily mechanised, gold mining techniques are used and surveillance should be instituted for this disease at sentinel mine locations.

## Supporting Information

S1 Text Supplementary Methods information. (DOCX)

## References

[1] Barros MB, de Almeida Paes R, Schubach AO. *Sporothrix schenckii* and Sporotrichosis. Clin Microbiol Rev. 2011 10;24(4):633–54. 10.1128/CMR.00007-11 21976602 PMC3194828

[2] Pijper AP, D B. An outbreak of sporotrichosis among South African native miners. Lancet. 1927 10:914–5.

[3] Lurie HI. Five unusual cases of sporotrichosis from South Africa showing lesions in muscles, bones, and viscera. British J Surg. 1963 5 1963;L(224):585–91. 10.1002/bjs.18005022406 13931772

[4] Dangerfield LF, Gear J. Sporotrichosis among miners on the Witwatersrand gold mines. S Afr Med J 1941 4:128–31.

[5] Vismer H, Hull PR. Prevalence, epidemiology and geographical distribution of *Sporothrix schenckii* infections in Gauteng, South Africa. Mycopathologia. 1997;137:137–43. 9368407 10.1023/a:1006830131173

[6] Kenyon C, Bonorchis K, Corcoran C, Meintjes G, Locketz M, Lehloenya R, et al A dimorphic fungus causing disseminated infection in South Africa. New Engl J Med. 2013 10;369(15):1416–24. 10.1056/NEJMoa1215460 24106934

[7] Marimon R, Gene J, Cano J, Trilles L, Dos Santos Lazera M, Guarro J. Molecular phylogeny of *Sporothrix schenckii* . J Clin Microbiol. 2006 9;44(9):3251–6. 16954256 10.1128/JCM.00081-06 PMC1594699

[8] Marimon R, Cano J, Gene J, Sutton DA, Kawasaki M, Guarro J. *Sporothrix brasiliensis*, *S*. *globosa*, and *S*. *mexicana*, three new *Sporothrix* species of clinical interest. J Clin Microbiol. 2007 10;45(10):3198–206. 17687013 10.1128/JCM.00808-07 PMC2045377

[9] Rodrigues AM, de Melo Teixeira M, de Hoog GS, Schubach TM, Pereira SA, Fernandes GF, et al Phylogenetic analysis reveals a high prevalence of *Sporothrix brasiliensis* in feline sporotrichosis outbreaks. PLoS Negl Trop Dis. 2013;7(6):e2281 10.1371/journal.pntd.0002281 23818999 PMC3688539

[10] Oliveira DC, Lopes PG, Spader TB, Mahl CD, Tronco-Alves GR, Lara VM, et al Antifungal susceptibilities of *Sporothrix albicans*, *S*. *brasiliensis*, and *S*. *luriei* of the *S*. *schenckii* complex identified in Brazil. J Clin Microbiol. 2011 8;49(8):3047–9. 10.1128/JCM.00255-11 21653757 PMC3147739

[11] Marimon R, Serena C, Gene J, Cano J, Guarro J. In vitro antifungal susceptibilities of five species of *Sporothrix* . Antimicrob Agents Chemother. 2008 2;52(2):732–4. 18039919 10.1128/AAC.01012-07 PMC2224726

[12] Ottonelli Stopiglia CD, Magagnin CM, Castrillon MR, Mendes SD, Heidrich D, Valente P, et al Antifungal susceptibilities and identification of species of the *Sporothrix schenckii* complex isolated in Brazil. Med Mycol. 2014 1;52(1):56–64. 10.3109/13693786.2013.818726 23964828

[13] Dias NM, Oliveira MM, Santos C, Zancope-Oliveira RM, Lima N. Sporotrichosis caused by *Sporothrix mexicana*, Portugal. Emerg Infect Dis. 2011 10;17(10):1975–6. 10.3201/eid1710.110737 22000393 PMC3310684

[14] O'Reilly LC, Altman SA. Macrorestriction analysis of clinical and environmental isolates of *Sporothrix schenckii* . J Clin Microbiol. 2006 7;44(7):2547–52. 16825378 10.1128/JCM.00078-06 PMC1489501

[15] Oliveira MM, Maifrede SB, Ribeiro MA, Zancope-Oliveira RM. Molecular identification of *Sporothrix* species involved in the first familial outbreak of sporotrichosis in the state of Espirito Santo, southeastern Brazil. Mem Inst Oswaldo Cruz. 2013 11;108(7):936–8. 10.1590/0074-0276130239 24141957 PMC3970636

[16] Reis RS, Almeida-Paes R, Muniz Mde M, Tavares PM, Monteiro PC, Schubach TM, et al Molecular characterisation of *Sporothrix schenckii* isolates from humans and cats involved in the sporotrichosis epidemic in Rio de Janeiro, Brazil. Mem Inst Oswaldo Cruz. 2009 8;104(5):769–74. 19820840 10.1590/s0074-02762009000500018

[17] Dixon DM, Salkin IF, Duncan RA, Hurd NJ, Haines JH, Kemna ME, et al Isolation and characterization of *Sporothrix schenckii* from clinical and environmental sources associated with the largest U.S. epidemic of sporotrichosis. J Clin Microbiol. 1991 6;29(6):1106–13. 1864926 10.1128/jcm.29.6.1106-1113.1991 PMC269953

