# Supplementary material for: An Outbreak of Lymphocutaneous Sporotrichosis among Mine-Workers in South Africa
Source: PLoS Negl Trop Dis. 2015 Sep 25;9(9):e0004096. doi: 10.1371/journal.pntd.0004096 (PMC4583532; doi:10.1371/journal.pntd.0004096)
Supplement: S1 Text — (DOCX) [file pntd.0004096.s001.docx]

*Submission to PLOS Neglected Tropical Diseases (Research Article)*

**An Outbreak of Lymphocutaneous Sporotrichosis among Mine-Workers in South Africa**

**Supplementary Methods**

Interventions before the outbreak investigation

Laboratory-confirmed lymphocutaneous sporotrichosis had first been diagnosed in two miners who had sought medical care in the private health-sector. Additional cases of suspected lymphocutaneous sporotrichosis were subsequently diagnosed on clinical grounds by the mine’s occupational health clinic among employees working at a reopened section of one of three operational mines (accessed through a single shaft). The exact date of reopening could not be established; contradictory information was obtained from workers and managers. Profuse growth of a white fungus, suspected to be *S. schenckii*, had also been reported by mine employees at several underground levels. Prior to the investigation, work on underground levels, where many miners with suspected sporotrichosis had been working prior to diagnosis, had been temporarily suspended. Targeted decontamination of working areas using a dilute hypochlorite solution had been instituted on levels that remained open. The mine company’s management team had also issued recommendations to miners to use personal protective equipment (PPE) consistently in areas with rotting timber material.

Specimen transport and processing

All clinical and environmental specimens were immediately refrigerated and transported on ice to the Mycology Reference Laboratory at the National Institute for Communicable Diseases (NICD) in Johannesburg, approximately 400 km away. Clinical specimens were inoculated onto 5 % horse blood agar and Sabouraud agar plates with and without cycloheximide (Diagnostic Media Products (DMP), Sandringham, South Africa) and incubated at 25 °C, 30 °C and 35 °C [34]. Isolates within the *S. schenckii* complex tolerate cycloheximide at a concentration of 0.25 % [4]. Selective procedures were also used to isolate *S. schenckii* from environmental samples (pers. comm. Dr. Hester Vismer). In brief, soil and wood samples were directly inoculated onto Sabouraud agar plates with and without cycloheximide (DMP). Soil and wood samples were also added to distilled water in a glass tube and vortexed for one minute after which the tubes were allowed to stand undisturbed for 15 to 30 minutes. This was repeated four times for each sample. One millilitre from the top of the suspension was then transferred with a pipette to Sabouraud agar plates with and without cycloheximide (DMP). All agar plates were incubated at 25 °C and examined daily for colonies that resembled *S. schenckii* complex. These colonies were then sub-cultured onto Sabouraud agar plates with cycloheximide and malt agar plates (DMP). All pathogens, which were isolated from clinical specimens, were identified to species-level and reported to the treating clinician at the time of the outbreak investigation.

Phenotypic identification

Slides cultures were set up for all colonies resembling *S. schenckii* sensu lato and incubated at 25 °C. The mould phase was examined microscopically. Isolates were converted to the yeast phase by incubating inoculated sheep blood and brain heart infusion agar plates (DMP) at 35 °C. Isolates were stored in water at room temperature following the outbreak investigation. Stored isolates were sub-cultured on Sabouraud and 5 % sheep blood and brain heart infusion agar plates (DMP) for further laboratory work.

Antifungal susceptibility testing

The minimum inhibitory concentration (MIC) for several antifungal agents (amphotericin B, voriconazole, itraconazole and posaconazole) was determined for isolates with confirmed species-level identification. MICs for fluconazole, flucytosine, caspofungin, micafungin and anidulafungin were also determined but these data are not reported here. MICs for all agents were determined using pre-prepared microbroth dilution panels (Thermo Fisher Scientific, Cleveland, Ohio, USA) as outlined in Clinical Laboratory Standards Institute (CLSI) M27-A3 [22]. The yeast phase was tested as this is the clinically relevant phase during disease; the inoculum was prepared to achieve a final concentration of 5 x 10^2^ – 2.5 x 10^3^ cells/mL. We have recently applied this method to testing the yeast phase of a novel dimorphic *Emmonsia* species [35]. MIC values were determined visually following 72 h of incubation at 35 ^o^C. For amphotericin B, the MIC was read as the lowest concentration that prevented any discernible growth (100 % inhibition). For the triazoles, the MIC was read at the point of 50 % inhibition compared to the drug-free growth control well. MICs were also determined by Etest (bioMérieux, Marcy ľEtoile, France) on RPMI 1640 plates containing 2 % glucose, as recommended by the manufacturer. The endpoints for amphotericin B were the same as the broth dilution test; however, triazole MICs were read at 80 % inhibition. The quality control isolates, *Candida parapsilosis* ATCC 22019 and *Candida krusei* ATCC 6258, were run on all days of testing; MICs were read after 24 h. Interpretive breakpoints or epidemiologic cut-off values (ECVs) were not available for *S. schenckii* and any antifungal agent.

Molecular identification and phylogenetic analysis

DNA extraction

The ZR Fungal/Bacterial DNA MiniPrep extraction kit (Zymo, Irvine, CA, USA) was used to obtain high quality DNA from all isolates that resembled *S. schenckii* complex by phenotypic methods. The concentration of DNA was measured using a nanophotometer (Implen GmbH, Munich, Germany).

PCR amplification of the internal transcribed spacer region of the ribosomal gene

The ITS region including the 5.8S rRNA operon was amplified with the ITS1 and ITS 4 primer pair [36]. A positive control of *Cryptococcus neoformans* (ATCC 34875 or ATCC 32608) was included. PCRs were made in 25 μL reactions comprising 2-9 ηg DNA, 1 U TrueStart Hot Start *Taq* DNA Polymerase ((Thermo Scientific, Waltham, USA), 1x buffer, 0.1 mM dNTPs, 1.6 mM MgCl_2_, 0.5 μM forward and 0.5 μM reverse primer. The PCR conditions were as follows: 96 ºC for 2 min, followed by 35 cycles of 94 ºC for 20 s, 55 ºC for 40 s and 72 ºC for 45 s, followed by an extension step of 72 ºC for 10 min and placed on hold at 4 ºC.

PCR amplification of the nuclear calmodulin gene

The nuclear CAL gene was sequenced using the CL1 and CL2A primer pair [3, 11]. PCRs were made in 25 μL reactions comprising 0.5-2 ηg DNA, 1 U TrueStart Hot Start *Taq* DNA Polymerase (Thermo Scientific, Waltham, USA), 1x buffer, 0.1 mM dNTPs, 1.6 mM MgCl2, 0.5 μM forward and 0.5 μM reverse primer. A positive control of *S. schenckii* ATCC 6243 was included. The following PCR conditions were used: 94 ºC for 15 min, followed by 40 cycles of 94 ºC for 20 s, 57 ºC for 30 s and 72 ºC for 90 s, followed by an extension step of 72 ºC for 15 min and placed on hold at 4 ºC.

PCR product visualisation and sequencing

Amplicons from both the ITS and CAL gene PCRs were run on a 2 % agarose (SeaKem, Lonza, USA) gel and visualised with UV fluorescence on a gel doc system (Vacutec, USA), together with a 100 bp molecular ladder (Fermentas, USA). Amplicons were purified using Exonuclease (10 units) and Shrimp alkaline phosphatase (2 units) (Thermo Scientific, Waltham, USA). Purified amplicons were subjected to a sequencing PCR; reactions consisted of the following: 2 μl 3.1 Big Dye, 3 μl 5x Buffer, 1 μl template, 1 μl 0.5 μM either forward or reverse primer and 13 μl sterile water. Sequencing PCR conditions consisted of 25 cycles of 95 ºC for 10 s, 55 ºC for 15 s and 60 ºC for 4 min. Sequencing PCR products were cleaned with 6 % Sephadex in columns. The samples were sequenced in a 3130 Sequencer (Applied Biosystems, Life Technologies Corporation, Carlsbad, CA, USA).

Sequence editing and alignment

Electropherograms were inspected and edited in Chromas Lite 2.01 (Technelysium, Brisbane, Australia). Consensus sequences were built in BioEdit sequence alignment editor (Ibis Biosciences, Carlsbad, CA, USA). All sequences were subjected to basic local alignment search tool (BLAST) analyses in GenBank (National Center for Biotechnology Information, National Library of Medicine, NIH, USA) for isolate identification. Pairwise sequence alignment of the ITS region was also performed using the pairwise sequence alignment tool in the Fungal Biodiversity Centre database (Centraalbureau voor Schimmelcultures - Royal Netherlands Academy of Arts and Sciences [CBS-KNAW], Utrecht, Netherlands) .

Phylogenetic analysis

Multiple consensus CAL gene sequences were aligned using the MAFFTonline (http://mafft.cbrc.jp/alignment/software/). CAL gene sequences of reference isolates (for each representative cryptic species) were obtained from GenBank based on data provided by Rodrigues et al and included in the phylogenetic analysis [6]. The following reference sequences were included (CAL GenBank accession numbers in parentheses): *S. brasiliensis* CBS 120339-T (AM116899), *S. schenckii* sensu stricto CBS 359.36-T (AM117437), *S. globosa* CBS 120340-T (AM116908), *S. luriei* CBS 937.72-T (AM747302), *S. mexicana* CBS 120341-T (AM398393) and *S. pallida* CBS 302.73-T (AM398396). Fungi within the genus *Sporothrix* are most closely related to the genus *Ophiostoma* and in particular, *Ophiostoma stenoceras* [2]. Therefore, *O. stenoceras* CBS 360.71-T (JX077127) was included. The following clinical strains from South Africa were included: *S. schenckii* sensu stricto IHEM 3787 (AM117435), *S. schenckii* (0392 Gauteng), *S. schenckii* (0336 Free State), *S. schenckii* (0335 Free State), *S. schenckii* (0377 KwaZulu Natal), *S. schenckii* (0389 KwaZulu-Natal), *S. schenckii* (MRC05), *S. schenckii* (MRC06) and *S. schenckii* (MRC50). *Grosmmania serpans* CBS 141.36-T (JN135300) was treated as the out-group taxon [6]. Sequences were exported as FASTA files and phylogenetic analysis was performed using the MEGA 6.0 software tool (Arizona State University, USA) by the neighbour-joining method. Evolutionary distances were computed using the Jukes-Cantor model. All positions containing gaps and missing data were eliminated.
